# Supplementary material for: Ensemble cryoEM elucidates the mechanism of insulin capture and degradation by human insulin degrading enzyme
Source: eLife. 2018 Mar 29;7:e33572. doi: 10.7554/eLife.33572 (PMC5910022; doi:10.7554/eLife.33572)
Supplement: Supplementary file 1. [file elife-33572-supp1.docx]

**Supplemental file 1 Data collection and structure refinement statistics**

|  | **IDE-Fab1-Insulin** | **IDE-Fab1-Aβ** | **IDE-Fab_H11-E_^f^** |  |
| --- | --- | --- | --- | --- |
| **Data Collection** |  |  |  |  |
| Beamline | APS-19ID | APS-19ID | APS 19ID |  |
| Wavelength (Å) | 0.9792 | 0.9792 | 0.9788 |  |
| Space group | P2_1_ | P2_1_2_1_2_1_ | P22_1_2_1_ |  |
| Cell dimension(Å) |  |  |  |  |
| a/b/c | 122/138/376 | 57/135/368 | 131 /242/311 |  |
| α/ β/ γ | 90.0/99.4/90.0 | 90.0/90.0/90.0 | 90.0/90.0/90.0 |  |
| Resolution (Å) | 100 – 3.95 | 100 – 3.50 | 50-3.80 |  |
| R_meas_ (%)^a^ | 24.7 (79.7)^e^ | 18.3 (78.1)^e^ | 34.3(94.1)^e^ |  |
| R_p.i.m_ (%)^b^ | 13.1 (42.4)^e^ | 10.3 (44.1)^e^ | 13.3 (36.4)^e^ |  |
| CC_1/2_^c^ | (0.583)^e^ | (0.530)^e^ | (0.846)^e^ |  |
| CC*^d^ | (0.858)^e^ | (0.832)^e^ | (0.957)^e^ |  |
| I/sigma | 5.0 (1.4)^e^ | 11.4 (2.4)^e^ | 8.0 (2.7)^e^ |  |
| Redundancy^g^ | 3.3 (3.3)^e^ | 3.0 (3.0)^e^ | 6.6(6.5)^e^ |  |
| Completeness (%) | 99.8 (99.5)^e^ | 96.6 (96.7) ^e^ | 100.0(100.0)^e^ |  |
| Unique reflections | 108388 | 36898 | 98658 |  |
| **Refinement** |  |  |  |  |
| R_work_ ^h^ | 0.24 | 0.23 | 0.22 |  |
| R_free_ ^i^ | 0.29 | 0.27 | 0.27 |  |
| No.atoms in protein | 86906 | 21517 | 47339 |  |
| B-factors |  |  |  |  |
| Protein | 111.9 | 79.2 | 77.2 |  |
| Substrate | 116.1 | 95.8 |  |  |
| r.m.s. deviations |  |  |  |  |
| Bond lengths (Å) | 0.002 | 0.002 | 0.003 |  |
| Bond angles (^o^) | 0.453 | 0.533 | 0.593 |  |
| Ramachandran Plot (%) | |  |  |  |
| Favorable region | 98.1 | 87.2 | 96.7 |  |
| Allowed region | 1.9 | 12.8 | 3.3 |  |
| Outliers | 0 | 0 | 0 |  |
| PDE code | 5WOB | 4M1C | 5UOE |  |
| ^a^ *R*_meas_ = Σ_hkl_ [n/(n-1)]^1/2^Σ_i_│I_hkl,I_ − <I_hkl_>│ ∕ Σ_hkl_ <I_hkl_>  ^b^ *R*_p.i.m._ = Σ_hkl_ [1/(n-1)]^1/2^Σ_i_│I_hkl,I_ − <I_hkl_>│ ∕ Σ_hkl_ <I_hkl_>  ^c^CC_1/2_ – Pearson correlation coefficient between random half-datasets - *ρ_x_,_y_=cov[(x,y)/(σ_x_σ_y_)]*  ^d^CC*=[2CC_1/2_/(1+CC_1/2_)]^1/2^  ^e^ the outer resolution shell. Values in parentheses indicate the highest resolution shell  ^f^ The elbow region of the Fab was engineered.  ^g^ *N*_obs_/*N*_unique_  ^h^*R*_work_ = Σ*_hkl_* \|\|F_obs_\| - *k* \|F_calc_\|\|/ Σ*_hkl_* \|F_obs_\|  ^i^ ***R***_free_, calculated the same as for **R**_work_ but on the 5% data excluded from the refinement calculation. | | | | |
