# Supplementary material for: Ensemble cryoEM elucidates the mechanism of insulin capture and degradation by human insulin degrading enzyme
Source: eLife. 2018 Mar 29;7:e33572. doi: 10.7554/eLife.33572 (PMC5910022; doi:10.7554/eLife.33572)
Supplement: Supplementary file 2. [file elife-33572-supp2.docx]

**Supplemental file 2 CryoEM data collection and processing statistic**

| **Protein** | **Insulin-bound IDE-Fab_H11-E_** | | | **IDE-Fab_H11-E_** | |
| --- | --- | --- | --- | --- | --- |
| **Data collection** | | | | | |
| Grids | Carbon lacey | Gold lacey nanowire | Gold lacey nanowire | Gold lacey  nanowire | Gold lacey manual |
| Vitrification Method | Manual  Plunger | Spotiton | Spotiton | Manual  Plunger | Spotiton |
| Microscope | Krios | Krios | Krios | Krios | Krios |
| Session name | 17mar04a | 17mar30a | 17may12a | 16sep10a | 17apr29a |
| Voltage (kV) | 300 | 300 | 300 | 300 | 300 |
| Magnification | 45,454 X | 45,454 X | 45,454 X | 45,454 X | 45,454 X |
| Tilt angle | 0 | 0 | 0 | 20-50 | 30 |
| Detector | K2 Summit | K2 Summit | K2 Summit | K2 Summit | K2 Summit |
| Recording mode | Counting | Counting | Counting | Counting | Counting |
| Dose rate (e^-^/s) | 6.9 | 7.4 | 7.1 | 7.9 | 6.8 |
| Pixel size (Å/pix) | 1.1 | 1.1 | 1.1 | 1.1 | 1.1 |
| Total dose (e^-^/Å^2^) | 63.1 | 67.6 | 64.5 | 72.3 | 61.8 |
| Number of frames | 50 | 50 | 50 | 50 | 50 |
| Total exposure time (s) | 10 | 10 | 10 | 10 | 10 |
| Set defocus range (µm) | 1.5 – 2.2 | 1.5 – 2.2 | 1.5 – 2.2 | 1.5 – 2.2 | 1.5 – 2.2 |
| **EM data processing** | | | | | |
| Number of micrographs | 625 | 582 | 1878 | 509 | 620 |
| Number of particles | 104,404 | 211,810 | 446,069 | 92,017 | 296,626 |
| Particles used for refinement | 41,231 | 40,196 | 56,965 | 29,621 | 155,971 |
| Total particles used for IDE dimer refinement | 148,392 | | | 151,868 | |
