# Supplementary material for: Ensemble cryoEM elucidates the mechanism of insulin capture and degradation by human insulin degrading enzyme
Source: eLife. 2018 Mar 29;7:e33572. doi: 10.7554/eLife.33572 (PMC5910022; doi:10.7554/eLife.33572)
Supplement: Supplementary file 3. [file elife-33572-supp3.docx]

**Supplemental file 3 CryoEM map and model refinement statistics**

| **Model Refinement** | **IDE-Fab****_H11-E_** | | | | | | | **Insulin-bound IDE-Fab****_H11-E_** | | | | | | |
| --- | --- | --- | --- | --- | --- | --- | --- | --- | --- | --- | --- | --- | --- | --- |
| Conformations | Open(O)/Open(O) | | O/partial Open (pO) | | pO/pO | | | partial closed (pC)/pC | | | | | | |
| Refinement mask | IDE dimer | Without  mask | IDE dimer | Without  mask | IDE dimer | Without | | IDE dimer | | IDE dimer | | Without mask | | |
| Total number of particles used for refinement | 24,425 | 24,425 | 110,.499 | 110,499 | 16,944 | 16,944 | | 148,392 | | 148,392 | | 148.392 | | |
| Map resolution  (FSC 0.143; Å) | 6.5 | 8.4 | 4.2 | 7.2 | 6.9 | 8.2 | | 3.7 | | 3.7 | | 4.1 | | |
| Global resolution (3DFSC) | 6.4 | 9.5 | 4.6 | 8.5 | 7.2 | 8.5 | | 4.1 | | 4.1 | | 4.6 | | |
| **Structure Refinement** | | | | | | | | | | | | |  | |
| Software | Phenix | Phenix | Phenix | Phenix | Phenix | | Phenix | | Phenix | | Phenix | | Phenix | |
| Cell-Dimensions |  |  |  |  |  | |  | |  | |  | |  | |
| a=b=c (Å) | 343 | 343 | 343 | 343 | 343 | | 343 | | 343 | | 343 | | 343 | |
| α=β=γ (º) | 90 | 90 | 90 | 90 | 90 | | 90 | | 90 | | 90 | | 90 | |
| Resolution (Å) | 343-6.5 | 343-8.4 | 343-4.2 | 343-7.2 | 343-6.9 | | 343-8.2 | | 351-3.7 | | 351-3.7 | | 351-4.1 | |
| CC_mask_ | 0.85 | 0.76 | 0.72 | 0.79 | 0.84 | | 0.86 | | 0.77 | | 0.78 | | 0.82 | |
| Protein residues | 1888 | 2744 | 1888 | 2743 | 1889 | | 2745 | | 1929 | | 1935 | | 2783 | |
| Total atoms | 15.410 | 21,840 | 15,412 | 21,836 | 15,407 | | 21,866 | | 15,727 | | 15,762 | | 22,192 | |
| Substrate (Insulin) | - | - | - | - | - | | - | | Ins A only | | Ins B only | | Ins A only | |
| R.m.s deviations |  | | | | | | | | | | | | | |
| Bond length (Å) | 0.007 | 0.006 | 0.006 | 0.008 | 0.007 | | 0.008 | | 0.005 | | 0.006 | | | 0.011 |
| Bond angle (º) | 1.180 | 1.29 | 1.18 | 1.386 | 1.166 | | 1.330 | | 1.041 | | 1.049 | | | 1.285 |
| Ramachandran (%) |  |  |  |  |  | |  | |  | |  | | |  |
| Favored | 96.27 | 93.6 | 96.22 | 93.56 | 96.44 | | 96.81 | | 97.39 | | 97.13 | | | 96.98 |
| Allowed | 3.73 | 6.4 | 3.78 | 6.44 | 3.56 | | 3.19 | | 2.61 | | 2.87 | | | 4.02 |
| Outliers | 0.0 | 0.0 | 0.0 | 0.0 | 0.0 | | 0.0 | | 0.0 | | 0.0 | | | 0.0 |
| Validation | | | | | | | | | | | | | | |
| MolProbity score | 1.80 | 2.11 | 1.67 | 1.90 | 1.70 | | 1.74 | | 1.30 | | 1.36 | | | 1.62 |
| Rotamer outliers (%) | 0.00 | 0.00 | 0.00 | 0.00 | 0.00 | | 0.00 | | 0.00 | | 0.00 | | | 0.00 |
| Clash score | 10.66 | 12.70 | 7.53 | 8.89 | 8.68 | | 10.61 | | 3.99 | | 4.18 | | | 6.20 |
