# Supplementary material for: Ensemble cryoEM elucidates the mechanism of insulin capture and degradation by human insulin degrading enzyme
Source: eLife. 2018 Mar 29;7:e33572. doi: 10.7554/eLife.33572 (PMC5910022; doi:10.7554/eLife.33572)
Supplement: Supplementary file 4. [file elife-33572-supp4.docx]

**Supplemental file 4 Distances and angles between center of mass of different domains of IDE cryoEM and crystal structures.**

|  | method | Chain | Buried surface (Å^2^) | Dihedral angle (^o^)^2^ | Center of mass (Å)^1^ | | | | | | | N-C Angle (^o^)^3^ |
| --- | --- | --- | --- | --- | --- | --- | --- | --- | --- | --- | --- | --- |
|  |  |  |  |  | D1-D4 | D2-D3 | N-C | D1-D3 | D2-D4 | D1-D2 | D3-D4 |  |
| hIDE - O/O | CryoEM | A-O | 668 | 34.8 | 51.6 | 36.7 | 43.3 | 52.0 | 52.7 | 31.1 | 31.1 | 83.1 |
|  |  | B-O | 727 | 35.5 | 53.0 | 37.8 | 44.4 | 53.9 | 52.5 | 31.6 | 30.2 | 85.0 |
| hIDE - pO/O | CryoEM | B-O | 706 | 39.5 | 55.1 | 38.5 | 45.7 | 54.6 | 53.3 | 31.2 | 30.2 | 88.6 |
|  |  | A-pO | 1514 | 25.1 | 35.7 | 32.7 | 33.5 | 44.1 | 46.3 | 31.0 | 31.0 | 61.1 |
| hIDE - pO/pO | CryoEM | A-pO | 1172 | 26.4 | 36.9 | 33.0 | 34.2 | 44.8 | 46.7 | 31.2 | 31.0 | 62.7 |
|  |  | B-pO | 964 | 27.0 | 38.8 | 34.5 | 35.9 | 46.0 | 47.7 | 31.3 | 30.5 | 65.7 |
| hIDE+insulin -pC/pC | CryoEM | A-pC | 2538 | 22.7 | 31.8 | 32.2 | 31.4 | 42.1 | 44.7 | 30.6 | 30.8 | 55.6 |
|  |  | B-pC | 2534 | 22.5 | 31.6 | 32.2 | 31.3 | 42.0 | 44.4 | 30.6 | 30.2 | 55.9 |
| hIDE-Fab_H11-E_  (5UOE) | X-ray | A-C | 2653 | 23.9 | 31.4 | 31.8 | 30.9 | 41.6 | 44.4 | 30.3 | 30.9 | 55.6 |
|  |  | B-C | 2619 | 23.7 | 31.4 | 31.7 | 30.9 | 41.5 | 44.2 | 30.2 | 30.6 | 55.5 |
| hIDE-Fab1 +insulin (5WOB) | X-ray | A-C | 2638 | 23.1 | 31.7 | 32.2 | 31.3 | 41.7 | 44.6 | 30.2 | 30.6 | 56.0 |
|  |  | B-C | 2643 | 24.2 | 31.7 | 31.9 | 31.1 | 41.7 | 44.5 | 30.2 | 31.0 | 55.8 |
| hIDE+Aβ (2G47) | X-ray | A-C | 2551 | 24.6 | 31.9 | 31.2 | 30.9 | 41.5 | 44.2 | 30.2 | 30.8 | 55.2 |
|  |  | B-C | 2585 | 24.6 | 31.2 | 31.9 | 30.9 | 41.4 | 44.3 | 30.3 | 30.8 | 55.2 |
| hIDE+Insulin (2WBY) | X-ray | A-C | 2570 | 23.7 | 31.3 | 31.7 | 30.9 | 41.5 | 44.2 | 30.4 | 30.6 | 55.4 |
|  |  | B-C | 2614 | 23.9 | 31.3 | 31.8 | 30.9 | 41.5 | 44.2 | 30.3 | 30.6 | 55.4 |
| hIDE (2JG4) | X-ray | A-C | 2597 | 24.7 | 31.5 | 31.8 | 31.0 | 41.5 | 44.4 | 30.3 | 30.8 | 55.6 |
|  |  | B-C | 2587 | 24.7 | 31.6 | 31.9 | 31.1 | 41.5 | 44.3 | 30.1 | 30.7 | 55.9 |
| rat IDE (3TUV) | X-ray | A-C | 2506 | 26.4 | 31.5 | 32.0 | 30.9 | 41.9 | 43.6 | 30.1 | 30.7 | 56.1 |
| E. coli pitrilysin  (1Q2L) | X-ray | A-C | 806 | 44.6 | 56.7 | 37.4 | 46.1 | 53.4 | 53.4 | 30.2 | 29.8 | 93.1 |

1. Human IDE-D1, aa residues 43-285; IDE-D2, aa residues 286-530; IDE-D3, aa residue 531-768; IDE-D4, 769-1011. Rat IDE domain definition is the same as that of human IDE. E. coli pitrilysin-D1, aa residues 24262; pitrilysin-D2, aa residues 263-506; pitrilysin-D3, aa residue 507-732; pitrilysin-D4, 733-960. Domain definition of pitrilysin is based on its structure alignment with human IDE.


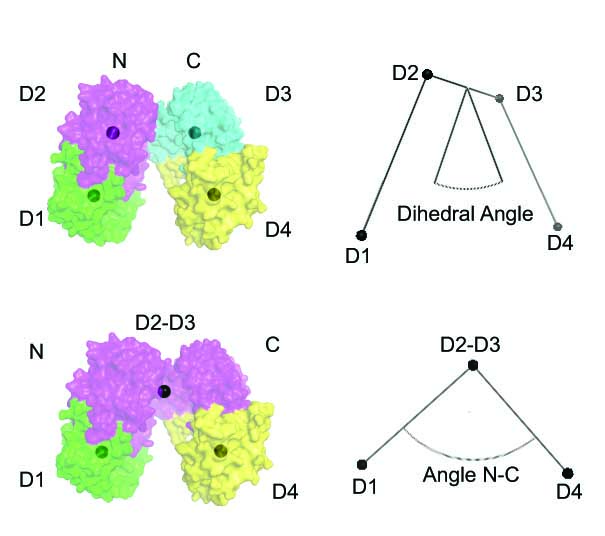


2.. Dihedral angle is defined by the angle between IDE-N domain and IDE-C domain. The axis of IDE-N is defined by the center of mass of IDE-D1 to that of IDE-D2 while that of IDE-C is by the center of mass of IDE-D3 to that of IDE-D4. The values of dihedral angles are negative and the absolute values are displayed.

3. N-C angle (angle between IDE-N and IDE-C) is defined by the center of mass of IDE-D1, the center of mass of IDE-D2+D3, and the center of mass of D4.
