# Supplementary material for: Ensemble cryoEM elucidates the mechanism of insulin capture and degradation by human insulin degrading enzyme
Source: eLife. 2018 Mar 29;7:e33572. doi: 10.7554/eLife.33572 (PMC5910022; doi:10.7554/eLife.33572)
Supplement: Transparent reporting form [file elife-33572-transrepform.pdf]

eLIFE
1st Floor
24 Hills Road
Cambridge CB2 1JP, UK
P 01223 855340
W elifesciences.org
T @elife

eLife's transparent reporting form

We encourage authors to provide detailed information *within their submission* to facilitate the interpretation and replication of experiments. Authors can upload supporting documentation to indicate the use of appropriate reporting guidelines for health-related research (see EQUATOR Network), life science research (see the BioSharing Information Resource), or the ARRIVE guidelines for reporting work involving animal research. Where applicable, authors should refer to any relevant reporting standards documents in this form.

If you have any questions, please consult our Journal Policies and/or contact us: editorial@elifesciences.org.

Sample-size estimation

- You should state whether an appropriate sample size was computed when the study was being designed
- You should state the statistical method of sample size computation and any required assumptions
- If no explicit power analysis was used, you should describe how you decided what sample (replicate) size (number) to use

Please outline where this information can be found within the submission (e.g., sections or figure legends), or explain why this information doesn't apply to your submission:

There are four major biophysical techniques employed in this study, cryoEM, macromolecular crystallography, small angle X-ray scattering (SAXS), and hydrogen deuterium exchange mass spectrometry (HDX-MS). For cryoEM and macromolecular crystallography, substantial efforts have been made to establish robust tools to refine the structures and evaluate the quality of experimental measurements in order to report the resolution of a giving structure and validity of the models. We have reported the software used in the key resource table within the star methods and data statistics in tables S1-3 for such analysis. The same is applicable to SAXS and HDX-MS. For HDX-MS, we have used the standard measurement and tools for the analysis are included in the method section. We have applied more specialized SAXS measurements, e.g., size exclusion chromatography-SAXS and time resolved SAXS. We have provided the experimental details in the data acquisition and analysis in the method section as well. All of the software package for our data analyses of SAXS and HDX-MS are also listed in the key resource table within the star methods.

Replicates

- You should report how often each experiment was performed
- You should include a definition of biological versus technical replication
- The data obtained should be provided and sufficient information should be provided to indicate the number of independent biological and/or technical replicates
- If you encountered any outliers, you should describe how these were handled
- Criteria for exclusion/inclusion of data should be clearly stated

- High-throughput sequence data should be uploaded before submission, with a private link for reviewers provided (these are available from both GEO and ArrayExpress)

Please outline where this information can be found within the submission (e.g., sections or figure legends), or explain why this information doesn't apply to your submission:

All four major biophysical tools, cryoEM, crystallography, SAXS, and HDX-MS require a large dataset with a lot of redundant data to fulfill the required statistical rigor. We have detailed the data statistics for crystallography in table 1 and those for cryoEM in table 2 and 3. For HDS-MS, SAXS, and the rest of biochemical measurements, the replications are indicated in method or in figure legends.

Statistical reporting

- Statistical analysis methods should be described and justified
- Raw data should be presented in figures whenever informative to do so (typically when N per group is less than 10)
- For each experiment, you should identify the statistical tests used, exact values of N, definitions of center, methods of multiple test correction, and dispersion and precision measures (e.g., mean, median, SD, SEM, confidence intervals; and, for the major substantive results, a measure of effect size (e.g., Pearson's r, Cohen's d)
- Report exact p-values wherever possible alongside the summary statistics and 95% confidence intervals. These should be reported for all key questions and not only when the p-value is less than 0.05.

Please outline where this information can be found within the submission (e.g., sections or figure legends), or explain why this information doesn't apply to your submission:

See the description above.

(For large datasets, or papers with a very large number of statistical tests, you may upload a single table file with tests, Ns, etc., with reference to sections in the manuscript.)

Group allocation

- Indicate how samples were allocated into experimental groups (in the case of clinical studies, please specify allocation to treatment method); if randomization was used, please also state if restricted randomization was applied
- Indicate if masking was used during group allocation, data collection and/or data analysis

Please outline where this information can be found within the submission (e.g., sections or figure legends), or explain why this information doesn't apply to your submission:

Not applicable or self-explanatory.

Additional data files ("source data")

- We encourage you to upload relevant additional data files, such as numerical data that are represented as a graph in a figure, or as a summary table
- Where provided, these should be in the most useful format, and they can be uploaded as "Source data" files linked to a main figure or table
- Include model definition files including the full list of parameters used
- Include code used for data analysis (e.g., R, MatLab)
- Avoid stating that data files are "available upon request"

Please indicate the figures or tables for which source data files have been provided:

All of the cryoEM and crystallographic data have been deposited to RCSB.
